# Investigating the Effects of Cultural-Mindset Priming on Evaluation of Job Performance Behaviors

**DOI:** 10.5964/ejop.v14i4.1617

**Published:** 2018-11-30

**Authors:** Vipanchi Mishra, Marcus Bost

**Affiliations:** a Department of Psychology, West Chester University of Pennsylvania, West Chester, PA, USA; b Human Capital Consultant, Deloitte, Philadelphia, PA, USA; Department of Psychology, Webster University Geneva, Geneva, Switzerland; Maynooth University, Maynooth, Ireland

**Keywords:** self-construal priming, collectivism, individualism, job performance, performance evaluation

## Abstract

Recent reviews of performance evaluation process and practices indicate that there is substantial variability in the structure and formalization of performance evaluations in organizations across cultures and call for further exploration of the role of cultural variables on the performance evaluation process. In the current study, we use self-construal priming procedures to evaluate the effects of cultural mindset on the performance evaluation process. Specifically, the effects of independent (individualistic) and interdependent (collectivistic) mindset priming on relative importance given to performance behaviors when making judgments of overall job performance was investigated. Participants first completed either independent (n = 87) or interdependent (n = 87) priming tasks by circling either I/me/my or we/us/our in a paragraph of text. Following this, they completed a managerial role-play exercise in which they read employee performance vignettes (manipulated on task, citizenship and counterproductive performance behaviors) and rated the overall performance of each employee. Rater policies were captured using regression analyses and relative weights placed on each performance behavior were computed. Results suggest that when making judgments of overall performance, as compared to raters primed with interdependence, raters primed with independence placed less weight on citizenship behaviors and higher weights on counterproductive performance behaviors. No significant differences were observed in the weights placed on task performance behaviors. Study limitations and implications for research are discussed.

Performance ratings play an important role in organizations; they are important not only in the traditional performance evaluation context but also in other employment practices, such as selection interviews and assessment centers. However, recent research has indicated that despite years of research and practice directed at improving the performance appraisal process in organizations, dissatisfaction with the process is at an all-time high (Adler et al., 2016). Specifically, it is estimated that more than 90% of managers, employees, and HR heads feel that their performance management processes do not deliver expected results, and many view their current processes as ineffective and/or inaccurate (Corporate Leadership Council, 2012). Given the ineffectiveness of this process, Adler et al. (2016) raise some controversial issues regarding whether performance rating systems should be eliminated or not. One reason Adler et al. (2016) identify for abolishing performance ratings is low levels of interrater agreement between raters on the same performance dimensions. Within a similar vein, Cho and Payne (2016) argue that cultural values may be related to the discrepancy observed in performance ratings. The purpose of the present study is to further explore the role of cultural values on evaluation of job performance behaviors and resulting performance ratings.

Recent research has indicated that rater values may play an important role in the performance evaluation and rating process. For example, in a recent study on understanding the effects of national culture on performance appraisal practices, Ng, Koh, Ang, Kennedy, and Chan (2011) found that power distance was related to performance rating leniency. Similarly, Mishra and Roch (2013) found that rater collectivism was associated with a preference for collectivistic ratees’ when assigning overall performance ratings. More recently, Mishra and Roch (2017) indicated that rater values of individualism and collectivism influenced the relative importance given to job performance behaviors when making decisions of overall performance, thus providing evidence that individual differences in rater values may influence performance appraisal ratings. However, while this research suggests that cultural values of individualism and collectivism may be responsible for the differential weighting of performance behaviors by raters when making overall performance ratings, it does not explain why this may be the case. Specifically, the mechanism through which a specific cultural value orientation may influence weighting of performance behaviors is not identified. In fact, Mishra and Roch (2017) note that future research should focus on identifying “why” raters with individualistic and collectivistic values differ in the weights placed on performance behaviors when making overall performance ratings. We believe that one way to understand the mechanism through which raters’ values influence the evaluation of job performance behaviors would be to use self-construal priming procedures and show that the pattern of rating differences become systematically greater when the construct of interest, i.e. individualism or collectivism is made accessible and salient to the raters, thus suggesting that these values are indeed responsible for the observed effects.

Priming studies involve the activation of relevant mental representations (values, beliefs, attitudes) in an unobtrusive manner in one phase of an experiment, and the unconscious, unintended effects of this activation are assessed in a subsequent phase (Bargh & Chartrand, 2000). Prior research has indicated that individuals in Western (individualistic) cultures view themselves as more independent whereas individuals in Eastern (collectivistic) cultures have a more interdependent view of self (Markus & Kitayama, 1991). This research has demonstrated that the degree to which a person thinks of himself or herself as relatively more independent or interdependent can be manipulated with priming tasks such as reading stories with independent versus interdependent themes (Trafimow, Triandis, & Goto, 1991), or circling singular (I, me, my etc.) vs. plural (we, us, our) pronouns in a paragraph of text (Gardner, Gabriel, & Lee, 1999). For example, Gardner et al. (1999) found that a pronoun circling task affected the degree to which participants endorsed individualistic values (like freedom and independence) and collectivistic values (like belongingness and friendship).

Priming studies can therefore temporarily focus participants’ attention on culture-relevant content (values, norms, beliefs, and attitudes), culture-relevant goals, and cultural-relevant cognitive styles. Thus, by comparing responses after individualism or collectivism is primed using self-construal primes, researchers can examine the extent to which psychological differences on dependent variables are actually due to the increased accessibility of individualistic or collectivistic mindset produced as a result of priming. While priming research has been done within the context of attention, visual perception and cognition (Choi, Connor, Wason, & Kahan, 2016; Haberstroh, Oyserman, Schwarz, Kühnen, & Ji, 2002; Kühnen & Oyserman, 2002), no research till date has investigated its effects in performance appraisal context. In the current study we investigate the influence of individual level cultural values on the evaluation of job performance behaviors (i.e. task, citizenship and counterproductive work behaviors) by priming individuals with independent and interdependent cultural mindsets and assessing how the resulting variations in context impact participants’ evaluation of job performance behaviors.

## Effects of Cultural Mindset Priming on Cognition

Self-construal has been broadly defined as “a constellation of thoughts, feelings and actions concerning one’s relationship to others, and the self as being distinct from others” (Singelis, 1994, p. 581). In relation to cultural differences, Markus and Kitayama (1991) proposed two distinct types of self-construals (*independent* and *interdependent* self-construals), which are based on individuals’ perceptions of how connected or separate they see themselves from others. Individuals with highly developed independent self-construals emphasize expressing their own thoughts and feelings, are interested in promoting their own goals, maintaining uniqueness and being direct in communication. The interdependent self, on the other hand, emphasizes fundamental connectedness of human beings to each other. Highly interdependent individuals tend to think of themselves and others as intertwined with each other rather than as separate entities. Originally, Markus and Kitayama (1991) had proposed that individuals in Western or individualistic cultures have predominantly independent self-construals, whereas individuals in Eastern or collectivistic cultures tend to have interdependent self-construals. However, subsequent researchers (e.g., Choi, Connor, Wason, & Kahan, 2016; Kühnen, Hannover, & Schubert, 2001; Suh, Diener, & Updegraff, 2008) have shown that individuals possess both independent and interdependent orientations of the self, and this sense of self influences cognition and behavior when individualistic or collectivistic value orientations are activated in a given situation. Specifically, Kühnen, Hannover, and Schubert (2001) developed a procedure called *mindset priming* to identify the mechanisms through which self-construal affects human cognitive processing. They proposed that self-construals activate different procedural modes of thinking that influences human cognition and behavior and referred to it as the semantic–procedural interface model of the self (Kühnen, Hannover, & Schubert, 2001). According to this model, views of the self are stored in a semantic framework and currently active conceptual information can alter a person’s procedural mode of thinking. The authors further proposed that the acquisition of independent self-knowledge relies on context-independent thinking procedures as it requires one to generalize self-descriptive features across the various contexts one encounters. On the other hand, defining the self in terms of relationships implies viewing identity as bound to the social context in which one encounters significant others. Thus, activation of independent self-construal induces context independent information processing modes, i.e. the tendency to process stimuli as if they were unaffected by the context, whereas, activation of an interdependent self-construal induces the tendency to process stimuli while paying attention to their relations to the entire field, i.e. context dependent information processing (Kühnen, Hannover, & Schubert, 2001). These procedural modes of thinking may then spill over and affect the way the person sees and or interacts with the world.

These predictions have been supported by many experimental studies that have used self- construal priming techniques (e.g., Choi, Connor, Wason, & Kahan, 2016; Haberstroh et al., 2002; Kühnen & Oyserman, 2002). These effects have been very well summarized by Oyserman and Lee (2008) in a meta-analytic review of the cultural priming literature. Overall, the results indicated that self-construal priming had a significant effect (mean weighted *d* = 0.34) on culture relevant content (such as values, self-concept, relationality) as well as cognition (context dependent vs. context independent thinking styles). With respect to self-concept, effect of priming was in hypothesized direction with an effect size of *d* = 0.26, thus suggesting that self-construal priming not only influences cognition, it also temporarily increases the accessibility of one self-concept versus the other. In the context of the current study, these findings may be useful in identifying the mechanisms through which cultural values influence raters’ overall performance judgments.

## Cultural Values, Priming and Evaluation of Job Performance Behaviors

The current conceptualization of job performance includes three aspects: task performance, citizenship performance, and counterproductive performance (Rotundo & Sackett, 2002). *Task performance* reflects behaviors that are consistent with completing job relevant tasks that relate to the core functioning of the organization (Murphy, 1989). *Citizenship performance* includes behaviors that are clearly related to organizational goals but do not necessarily contribute to the core functioning of the organization (Borman & Motowidlo, 1993) and are referred to as organizational citizenship behaviors (OCBs). The third domain called *counterproductive performance* represents behaviors that prevent or deter an organization from achieving its goals such as theft, drug abuse, etc. (Robinson & Bennett, 1995) and are referred to as counterproductive work behaviors (CWBs). There has been empirical evidence suggesting that these three job performance domains are empirically distinct (Dalal, 2005; Rotundo & Sackett, 2002; Sackett, Berry, Wiemann, & Laczo, 2006). Prior research has indicated that when making judgments of overall employee performance, managers take into account all three types of performance behaviors (e.g., Mishra & Roch, 2017; Rotundo & Sackett, 2002). Furthermore, cross-cultural research has indicated that managers from collectivistic versus individualistic countries differ in the importance they give to performance related behaviors when deciding on a performance bonus or compensation.

For example, Beatty, McCune, and Beatty (1988) used a policy capturing approach to compare compensation decisions made by managers in Japan and USA. Results indicated that managers from the United States placed the most importance on job performance whereas managers from Japan considered factors such as job worth and organizational commitment to be relatively more important than job performance in their decision- making policy. Similarly, Zhou and Martocchio (2001) examined the extent to which four variables (work performance, relationship with coworkers, relationship with managers, and personal needs) affected Chinese and American managers’ compensation award decisions (about bonus amounts and non-monetary recognition). Results showed that as compared to American managers, Chinese managers put less emphasis on work performance when making performance bonus decisions. Secondly, when making non-monetary recognition decisions, as compared to American managers, Chinese managers placed more importance on employees’ relationship with managers and coworkers, thus giving higher ratings to employees who had better interpersonal relationships with the managers. In another study, Hu, Hsu, Lee, and Chu (2007) compared the reward allocation decisions of managers in Taiwan and USA and found that the mean gap of the Taiwanese managers’ bonus allocation for close affect subordinates and distant affect subordinates was larger than that of the U.S. managers, suggesting that Taiwanese managers gave higher bonuses to subordinates with closer affective relationships, whereas, American managers gave higher bonuses to subordinates with higher task performance.

In contrast to task performance, studies on managers’ perceptions of organizational citizenship behaviors suggest that OCBs are perceived as in-role behaviors in collectivistic cultures. For example, Lam, Hui, and Law (1999) found that supervisors and employees from collectivistic cultures (Japanese and Hong Kong) tended to view the OCB dimensions of *courtesy* and *sportsmanship* as required parts of their subordinates’ jobs, while their individualistic counterparts (Americans and Australians) did not. In addition, Moorman and Blakely (1995) found that individuals holding collectivistic values were more likely to perform OCBs than those holding individualistic values; these results were significant and remained robust even after controlling for procedural justice and common method variance. Similarly, VanDyne, Vandewalle, Kostova, Latham, and Cummings (2000) found that individual differences in collectivism predicted OCBs measured six months later, also suggesting that collectivistic individuals are more likely to perform OCBs. These findings imply that collectivistic supervisors may weigh citizenship behaviors more heavily because they may see them not as discretionary but as important behaviors necessary to complete work related tasks.

In contrast to OCBs and task performance, very limited research has looked at the relative importance given to counterproductive behaviors in overall performance ratings in cultural contexts. In one study of managers across United States and Canada, Rotundo and Sackett (2002) found that counterproductive behaviors were given equal weight as task performance in overall performance ratings by supervisors (β = .47 and β = -.47), whereas citizenship behaviors accounted for very little variance (β = .26). In a second study focused on counterproductive behaviors, Rotundo and Xie (2008) found that managers from China placed less weight on counterproductive performance as compared to task performance (β = -.43 and β = .65 respectively). More recently, Mishra and Roch (2017) found that as compared to raters with individualistic values, raters with collectivistic values gave less importance to task and CWBs, and more importance to OCBs when making overall performance decisions. One reason for these findings could be that since collectivistic individuals are focused on maintaining the harmony of the workgroups (Jackson et al., 2006), they may be more likely to focus on positive behaviors and less likely to be critical of negative behaviors displayed by the workgroup members; hence giving less importance to CWBs than individualistic raters when making overall performance decisions.

Thus, based on the country level and individual level results described above, several conclusions can be drawn regarding the effects of individual differences in cultural values on performance evaluation process. Firstly, because collectivists favor the goal of promoting group welfare (Jackson et al., 2006), it may be possible that citizenship behaviors that are directed towards the benefit of others are perceived by collectivistic raters to be more important in making decisions of overall performance as compared to individualistic raters. Furthermore, prior research suggests that in order to preserve their relationships, collectivistic individuals often choose not to be critical of others’ undesirable behaviors (Bond, 1994); hence it may be possible that they may not give as high importance to CWBs as individualistic raters. On the contrary, because individualists emphasize personal outcomes and value competitiveness and achievement (Triandis, 1993) when judging the performance of employees, the primary concern of managers in individualistic cultures may be on the accomplishment of performance related tasks and achievement of organizational performance goals (e.g., Hu, Hsu, Lee, & Chu, 2007; Mishra & Roch, 2013; Rotundo & Xie, 2008; Zhou & Martocchio, 2001). Consequently, individualistic raters may be more likely to give higher importance to task and counterproductive performance behaviors when making judgments of overall performance.

If indeed individualism and collectivism are responsible for cross-cultural differences observed in the importance given to different performance behaviors in these studies, the use of cultural-mindset priming should make these effects more salient. Specifically, activation of *interdependent self-construal* should make collectivistic values more salient, evoking a context dependent thinking style. Therefore, these raters would be more likely to consider contextual factors when evaluating overall performance of the ratees and may therefore place less importance on task performance than those primed with an independent self-construal. On the other hand, those raters primed with an *independent self-construal* may be less likely to focus on the workgroup context due to the activation of context independent thinking styles and may therefore place more importance on task performance behaviors as compared to unprimed raters.

Thus, by combining the research on cultural-mindset priming and the research indicating cultural differences in the importance given to different performance behaviors, the following hypothesis is proposed:

Hypothesis: When making judgments of overall employee performance, as compared to raters primed with an independent self-construal, raters primed with an interdependent self-construal will 1a) give higher weights to OCBs 1b) lower weights to CWBs 1c) lower weights to task performance behaviors.

## Methods

### Participants

Participants were 174 undergraduate students from a suburban University located in the Northeastern region of the United States who received 1 research credit for participation in the study. Sixty-three percent were women. Majority of the participants were Caucasian (67.3%), 11% were African American, 8.3% were Asian American, and 3.3% identified themselves as belonging to the Other ethnicity. Most participants (93.7%) were between the ages of 18-22 years; 63% reported having performance appraisal experience.

### Measures

#### Employee Performance Profiles

We adapted nine customer service employee profiles and associated expert ratings of these profiles from Mishra and Roch (2017) for the purpose of this study. Each profile included a description of employees’ task, citizenship and counterproductive behaviors at work, even though they were not labeled as such for the participants. Each profile was independent of the others and consisted of two task performance behaviors, two citizenship performance behaviors, and two counterproductive performance behaviors, for a total of nine independent behavioral profiles of customer service representatives. Presentation of performance behaviors was counterbalanced across the profiles (see sample profile in the Appendix).

#### Priming Tasks

The “pronoun circling task” developed by Gardner, Gabriel, and Lee (1999) was used in the current study. Participants were provided with a short paragraph about a trip to the beach (adapted from Oyserman, Sorensen, Reber, & Chen, 2009) and asked to circle the pronouns as they read through the paragraph. In the independent prime condition, participants were asked to circle singular pronouns such as “I”, “Me”, “My” etc. In the interdependent prime condition, participants were asked to circle plural pronouns such as “We”, “They”, “Us” etc.

#### Self-Construal Measures

For manipulation check in each priming condition, pre and post priming self-construal orientations were measured using Singelis (1994) independent (Cronbach *α* = .81) and interdependent self-construal measures (Cronbach *α* = .77). The independent self-construal measure is comprised of 15 items. An example item is “I am comfortable with being singled out for praise or rewards.” The interdependent self-construal measure is comprised of 15 items. An example item is “My happiness depends on the happiness of those around me.”

#### Overall Performance Rating

Participants rated the overall level of performance of each of the nine employees using a seven-point Likert scale, anchored from *1 = low overall performance* to *7 = high overall performance*.

#### Demographic Characteristics

Participants were asked to report their age, gender, ethnicity, major, year in school, work hours, and previous performance rating experience.

### Procedure

The study was conducted in a lab setting. After reading and signing the consent form, participants first completed a questionnaire that included measures of baseline self-construal that were embedded within other measures of individual differences not related to this study. They were then randomly assigned to one of the two priming conditions. In the independent prime condition, participants were asked to circle singular pronouns such as “I”, “Me”, “My” etc. In the interdependent prime condition, participants were asked to circle plural pronouns such as “We”, “They”, “Us” etc. Following the priming task, each participant was asked to assume the role of a store manager and asked to review performance incidents in the last six months of nine customer service employees working in their store. Upon review of each profile, they were asked to provide an overall performance rating to each employee. The presentation order of profiles was rotated across participants. Participants then completed a post priming self-construal measure and a demographic questionnaire. The study sessions lasted approximately 45 minutes.

## Results

Table 1 provides the means, standard deviations and inter-correlations among study variables.

**Table 1:** Means, Standard Deviations and Inter-Correlations Among Study Variables by Priming Condition

Variable	*M*	*SD*	1	2	3	4	5	6	7	8	9	10	11	12	13
1. Independence T1	5.18^a^	0.73	(.77)	.93**	.02	.02	-.05	.09	.08	.04	.01	-.22*	-.04	-.10	-.13
2. Independence T2	5.29^b^	0.75	.96**	(.81)	.12	.15	-.07	.07	.06	-.05	-.05	-.25*	-.07	-.17	-.21
3. Interdependence T1	4.83^c^	0.61	.15	.12	(.73)	.94**	-.03	-.03	-.16	-.08	-.05	-.10	-.12	-.17	-.18
4. Interdependence T2	4.91^d^	0.68	.19	.17	.95**	(.76)	-.10	-.02	-.11	-.09	-.01	-.17	-.16	-.21	-.24*
5. HA1	2.40	1.05	-.06	-.10	-.14	-.15	–	.14	.19	.29**	.39**	.35**	.26*	.35**	.36**
6. TA1	4.94	0.97	.06	.04	.06	.10	.03	–	.52**	.08	.19	.10	-.08	.24*	.11
7. AL1	3.49	1.09	-.19	-.15	-.09	-.06	.10	.26*	–	.15	.12	.08	-.01	.16	.07
8. CH1	4.76	1.19	.08	.07	-.12	-.09	.20	.28**	.01	–	.62**	.40**	.44**	.41**	.46**
9. DE1	5.00	1.18	.11	.08	-.12	-.11	.34**	.35**	.19	.50**	–	.49**	.37**	.30**	.45**
10. JE1	4.76	1.18	-.06	-.06	-.08	-.10	.13	.11	.36**	.12	.40**	–	.49**	.44**	.38**
11. JO1	3.51	1.23	.03	.06	-.16	-.10	.27*	.23*	.14	.55**	.30**	.17	–	.30**	.56**
12. RE1	2.80	1.13	.10	.08	.08	.10	.35**	.23*	.25*	.24*	.38**	.44**	.35**	–	.33**
13. JA1	4.46	1.07	.05	.05	.11	.16	.16	.26*	.19	.34**	.43**	.25*	.16	.35**	–

*Note.* HA1, TA1, AL1, CH1, DE1, JE1, JO1, RE1, JA1- overall performance rating given to nine performance profiles respectively. Values on the top diagonal are for participants primed with interdependent (collectivistic) mindset and values on the bottom of the diagonal are for participants primed with independent (individualistic) mindset.

^a,b^Mean before and after ratings of independent self-construal in independent prime condition.

^c,d^Mean before and after ratings of interdependent self-construal in interdependent prime condition.

*p < .05. **p < .01. ***p < .001.

### Manipulation Check

Paired sample *t-tests* on pre-post scores on self-construal measures were conducted. As expected, results indicated an increase in the independent self-concept after priming, *M*_D_ = .11, *SD* = .20, *t*(86) = 5.25, *p* < .001, for independent prime condition and an increase in interdependent self-concept, *M*_D_ = .08, *SD* = .20, *t*(86) = 3.24, *p* < .01, in the interdependent prime condition.

### Computation of Relative Regression Weights

In order to determine the relative importance given by each rater to each of the performance behaviors (task, OCB and CWB) when making an overall performance rating decision, we first computed relative regression weights (Johnson, 2000) for each rater. For this purpose, individual regression analyses were conducted for each rater, with mean expert ratings of task, OCB and CWBs for each profile as predictors and overall performance ratings across the nine profiles given by the rater as the dependent variable. This resulted in an overall estimate of relative regression weights placed by each rater on each of the performance behaviors when making an overall performance rating decision. These relative regression weights were then used in the primary analyses described below.

### Priming Effects on Weights Placed on Performance Behaviors

**Figure 1:**
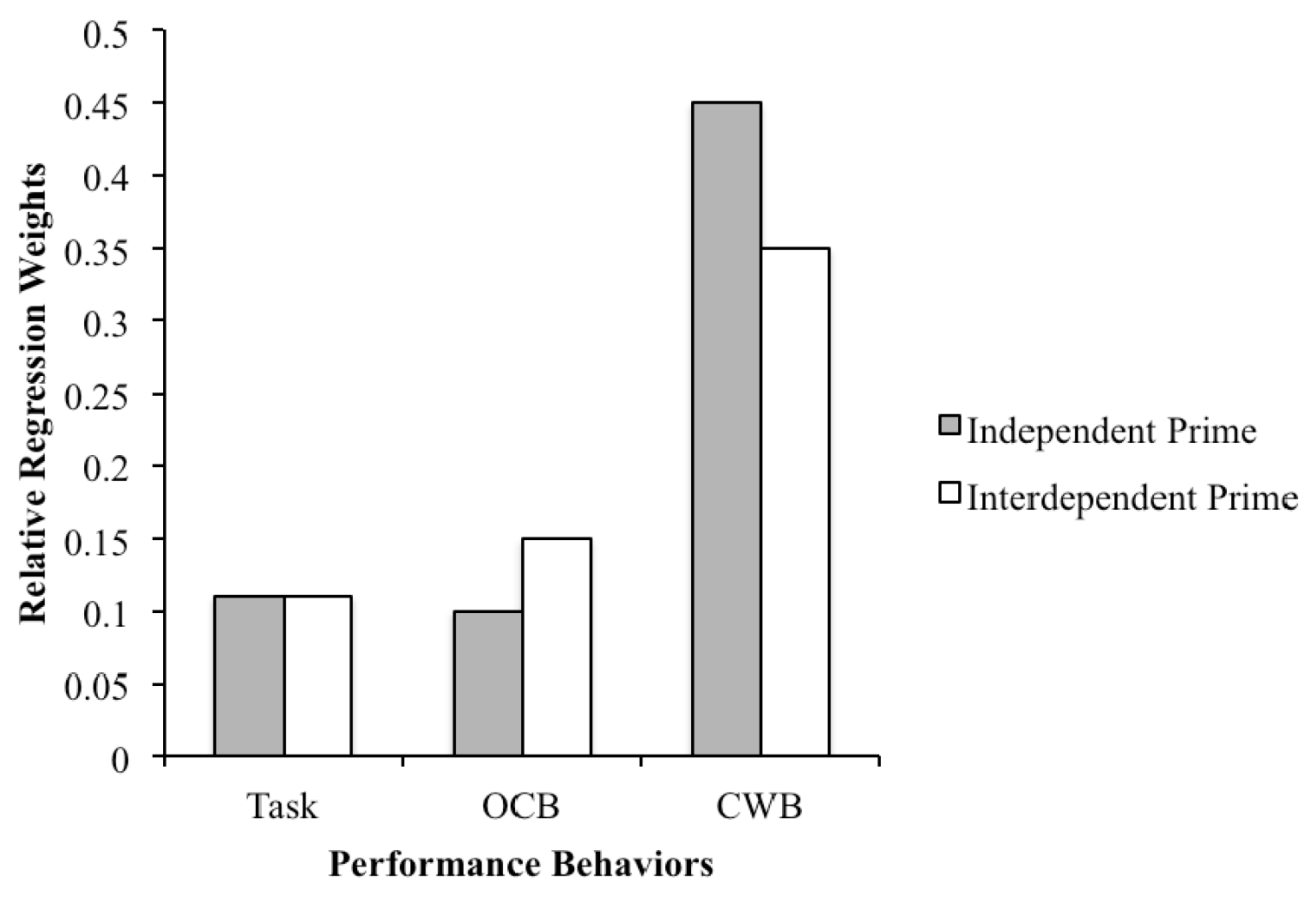
Relative regression weights placed on performance behaviors by priming condition.

A MANOVA with relative regression weights placed on task, citizenship and counterproductive behaviors for each rater as the dependent variables and priming condition as the between subject factor was conducted. Participants’ performance appraisal experience and presentation order of profiles were entered as covariates in the analysis. Results indicated a significant multivariate main effect of priming condition on weights placed on task, citizenship and counterproductive performance behaviors (Wilks lambda = .93, (*F*(3, 168) = 4.10, *p* < .01, partial η^2^ = .07). Performance appraisal experience (Wilks lambda = .97, (*F*(3, 168) = 1.72, *n.s.*) and presentation order of profiles (Wilks lambda = .98, (*F*(3, 168) = .74, *n.s.*) did not have any significant effect on relative weights placed on performance behaviors. Follow up analysis indicated a significant univariate effect of priming condition on weights placed on citizenship performance behaviors (*F*(1, 170) = 6.71, *p* < .05, partial η^2^ = .04). As compared to raters primed with an independent mindset (*M* = .10, *SD* = .11), raters primed with interdependent mindset placed higher weights (*M* = .15, *SD* = .14) on citizenship performance behaviors when assigning overall performance ratings, thus Hypothesis 1a was supported. A significant univariate effect of priming condition on weights placed on counterproductive performance behaviors (*F*(1, 179) = 9.54, *p* < .01, partial η^2^ = .05) was also obtained. As compared to raters primed with an interdependent mindset (*M* = .35, *SD* = .20), raters primed with independent mindset placed higher weights (*M* = .45, *SD* = .22) on counterproductive performance behaviors when assigning overall performance ratings, thus Hypothesis 1b was also supported. Lastly, no significant univariate effect of priming condition on weights placed on task performance behaviors (*F*(1, 179) = .11, *n.s*)*.* was found. Thus, Hypothesis 1c predicting that raters primed with an independent mindset will place more weight on task performance behaviors than raters primed with interdependence was not supported. A comparison of relative weights placed on all three types of job performance behaviors across the two priming conditions is presented in Figure 1.

## Discussion

The results show that the importance placed on different types of performance behaviors when making overall performance judgments may depend on the situation, i.e. relative importance given to performance behaviors may differ depending on whether the situation activates an individualistic (independent) or a collectivistic (interdependent) mindset. Not only has previous research tended to assume that cultural values assessed on the country level translate to the individual rater but also, even more importantly, no research has simultaneously investigated differences in relative importance given to task, OCB and CWBs in judgments of overall performance from a cultural-mindset or *state* perspective. In accordance with the semantic-procedural interface model (Kühnen, Hannover, & Schubert, 2001), we were able to demonstrate that priming individuals with independent vs. interdependent mindset influences their cognitive processing style, consequently influencing their judgments of overall job performance in a performance appraisal context. Our findings therefore shed some light on “why” there are differences observed in the evaluation of job performance behaviors by managers with individualistic or collectivistic values as suggested by prior research (e.g., Mishra & Roch, 2017). These results add to the performance appraisal literature in two ways: a) in part, our study replicates the results observed at the cultural level, which has indicated cross-cultural differences in managers’ ratings of contextual performance behaviors when it comes to making decisions related to employee bonus, or compensation; b) our results demonstrate that situational characteristics may make the effects of cultural values on behavioral outcomes more salient, thus presenting an alternative method for researchers and practitioners interested in evaluating the effects of cultural values on workplace behaviors.

Specifically, our results indicate that in comparison to raters primed with independent (individualistic) mindset, raters primed with an interdependent (collectivistic) mindset gave significantly higher weights to citizenship behaviors when making judgments of overall performance. These findings are in accordance with previous research on mindset priming (Kühnen, Hannover, & Schubert, 2001), which has shown that priming individuals with a collectivistic mindset increases their context-dependency in different types of social situations. Prior research has shown that activation of an interdependent self-construal induces context dependent information processing, i.e. the tendency to process stimuli while paying attention to their relations to the entire field (Choi, Connor, Wason, & Kahan, 2016; Kühnen, Hannover, & Schubert, 2001; Kühnen & Oyserman, 2002). The present study extends this phenomenon to the organizational context by demonstrating that increasing accessibility to a context dependent thinking style increases raters’ sensitivity to contextual behaviors and most likely that is why they tend to place higher weights on OCBs when making overall performance judgments. Because organizational citizenship behaviors are not directly related to job performance but are related to the broader organizational context, it may be that when individuals were primed with an interdependent mindset it may have resulted in an increased focus on contextual performance behaviors resulting in higher weights placed on OCBs when assigning an overall performance rating as compared to those primed with an independent mindset. This suggests that when the characteristics of the situation require collective functioning of the group and interdependence among group members, OCBs may become more salient to the raters. This finding has implications for the practice of performance appraisal in organizations. For instance, it may be possible that in a team-based setting, where individual rewards and performance are tied to team performance, managers may perceive behaviors directed towards improvement of group performance more positively. Hence, they would be more likely to reward and give higher importance to OCBs when making judgments of overall performance.

Secondly, results indicate that raters primed with an interdependent mindset gave significantly lower weights to CWBs when making judgments of overall performance as compared to those primed with an independent mindset. Previous research has shown that saving face for oneself and that of others is so deeply rooted in collectivistic individuals that they often choose not to be critical of others’ undesirable behaviors (Bond, 1994); hence it may be that activation of a context dependent mindset may have increased the focus of the raters on positive behaviors and consequently they may have placed less importance on negative behaviors. On the contrary, given that individualists are more likely to engage in behaviors that benefit themselves (Triandis, 1993), it may be that when raters were primed with an independent mindset they were more likely to focus on behaviors that were directly related to the achievement of performance goals. Because CWBs inhibit the attainment of task performance, these behaviors may have been given significantly higher weights by raters primed with an independent mindset.

Finally, our findings indicate that raters in both priming conditions gave similar weights to task performance behaviors when making judgments of overall performance. One reason for these findings could be that because task performance is more of an objective indicator of one’s performance on a job as compared to citizenship or counterproductive performance, raters may have given these behaviors similar importance in ratings of overall job performance irrespective of how they were primed. Taken together, these findings are an initial step in helping us understand the role of the cultural context on evaluation of performance behaviors, however, further replication using managerial samples is necessary to advance the literature in the field.

### Study Limitations

One of the limitations of the present study was that this study utilized a paper people approach to investigate the effects of self-construal priming on weights placed on job performance behaviors, which has been criticized for its lack of applicability in field settings (Murphy, Herr, Lockhart, & Maguire, 1986). However, in a comparison of paper people vs. behavioral observation studies, Murphy, Herr, Lockhart, and Maguire (1986) found that the difference in these samples is of little practical importance. Thus, although there is a need for replication of this study using either direct observation or actual employee data, the use of paper people should not be a topic of significant concern. Another potential limitation of this study is that it was conducted in a laboratory with undergraduate student participants. It can be argued that practicing managers would have a better understanding of the position and the job itself and hence would provide ratings that are different from those given by undergraduate students. However, much research in the field of organizational behavior has suggested that student participants in the lab provide very similar and sometimes identical results to managerial field samples (e.g., Dipboye, 1990). However, as with any research study, replication is needed.

### Directions for Future Research

The results of the current study provide several directions for future research. One limitation of this study was that it did not manipulate the purpose of appraisal. Prior research has shown that individual differences in rating behaviors are likely to be observed as a function of the purpose of appraisal (DeNisi & Williams, 1988). Thus, it may be possible that activation of culture specific mindset may have different effects on overall performance ratings and relative weights placed on job performance behaviors as a function of appraisal purpose. For instance, raters primed with an individualistic mindset may give significantly higher weights to counterproductive work behaviors and task performance behaviors when the purpose of appraisal is to promote employees, whereas raters primed with a collectivistic mindset may give more importance to citizenship performance behaviors as they prefer to reward those who contribute towards group well-being.

Secondly, the current study only focused on individualism/collectivism as a source of cultural value differences among raters. There are several other value dimensions such as power distance, long-term orientation vs. short-term orientation, masculinity vs. femininity etc. (Hofstede, 2001), which were not considered in the current study. Future research should look at the effects of other cultural mindsets on raters’ judgment policies to give a more comprehensive understanding of the effects of culture on performance judgments. For instance, there is some indication in the literature that in cultures characterized by high power distance, unequal distribution of power is preferred as a result of which managerial decisions are autocratic in nature and employee participation is discouraged (Mendonca & Kanungo, 1996). Thus, individuals primed with high power distance may be more direct and forthright during the performance appraisal process and may be excessively concerned with task performance behaviors when making judgments of overall performance. These differences in managerial behavior can influence the practice of performance appraisal as well.

### Practical Implications

Our findings indicate that situational characteristics may influence the extent to which raters give weights to specific performance behaviors when determining overall performance ratings. One implication of these findings could be to develop rater training programs that are directed towards creating a common frame of reference for raters when evaluating performance behaviors, thus eliminating situational biases when it comes to evaluating performance dimensions (Roch, Woehr, Mishra, & Kieszczynska, 2012). Secondly, understanding how cultural values influence rating behavior may help us to better understand the influence of individual differences, such as motivation, personality, etc. on performance ratings in a specific cultural context. For example, understanding how managers’ cultural values influence ratings may give upper management a better perspective of the ratings and allow for higher quality decisions among subordinates of different managers. Thirdly, these findings have implications for the design of employee recognition programs. Since activating a collectivistic mindset increases preference for OCBs, organizations can create reward programs such that employees exhibiting OCBs in team situations are rewarded thus increasing overall team performance in the long run.

In summary, these results add to the performance appraisal research literature by demonstrating that in addition to the direct *trait like* effects of cultural values (i.e. measurement of individualism and collectivism) on overall performance ratings, there are possible *state like* effects of cultural values on relative weighting of performance behaviors as well, i.e. situational characteristics may make the effects of cultural values more salient. These findings may help explain why there are discrepancies in managerial performance ratings of the same dimensions and the resulting unhappiness from the performance appraisal process.

## Figures and Tables

**Figure:**
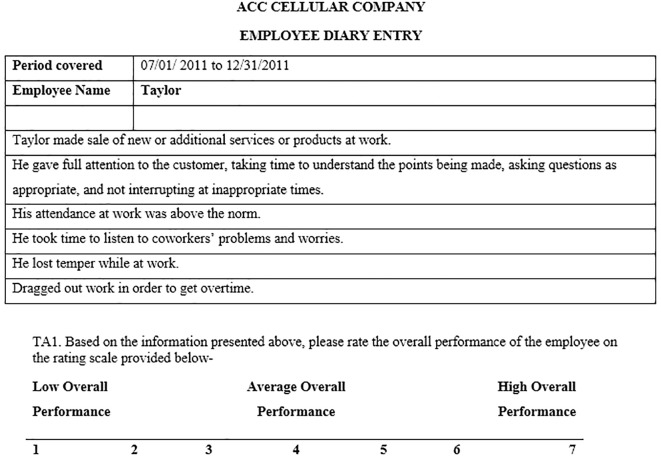
(Adapted from Mishra & Roch, 2017).

## Appendix: Employee Performance Evaluation

Imagine that you work at ACC Cellular Corporation as a Manager of Customer Service Department. Given below are performance profiles of customer service employees working in your store. These performance profiles contain list of behaviors recorded in the employee diary by the employees’ direct supervisor during the last six months at work. These diary entries describe actions and behaviors of employees that relate to job performance. Information about three different groups of behaviors is presented in these diary entries:

1. information about performing the duties of a customer service employee
2. information about how each employee contributes to the social and psychological environment of the company
3. information about each employee’s personal discipline

Please read each diary entry carefully and use only the information that is provided within each profile to evaluate each employee’s **overall job performance. **Use the seven-point rating scale at the end of the page to make your rating. A ***rating of 1 indicates low overall performance*** and a ***rating of 7 indicates high overall performance.***
